# A challenge to all. A primer on inter-country differences of high-need, high-cost patients

**DOI:** 10.1371/journal.pone.0217353

**Published:** 2019-06-19

**Authors:** Marit A. C. Tanke, Yevgeniy Feyman, Enrique Bernal-Delgado, Sarah R. Deeny, Yuichi Imanaka, Patrick Jeurissen, Laura Lange, Alexander Pimperl, Noriko Sasaki, Michael Schull, Joost J. G. Wammes, Walter P. Wodchis, Gregg S. Meyer

**Affiliations:** 1 Harvard T.H. Chan School of Public Health, Boston, Massachusetts, United States of America; 2 Radboudumc, Nijmegen, the Netherlands; 3 Commonwealth Fund Harkness Fellowship, New York, New York, United States of America; 4 Boston University School of Public Health, Boston, Massachusetts, United States of America; 5 Institute for Health Sciences in Aragon, Zaragoza, Aragon, Spain; 6 The Health Foundation, London, United Kingdom; 7 Kyoto University Graduate School of Medicine, Kyoto, Japan; 8 OptiMedis AG, Hamburg, Germany; 9 Sunnybrook Research Institute, Toronto, Ontario, Canada; 10 University of Toronto, Toronto, Ontario, Canada; 11 Institute for Better Health, Trillium Health Partners, Mississauga, Ontario, Canada; 12 Partners Healthcare System, Boston, Massachusetts, United States of America; 13 Harvard Medical School, Boston, Massachusetts, United States of America; University Complutense of Madrid, SPAIN

## Abstract

**Background:**

Across countries, a small group of patients accounts for the majority of health care spending. These patients are more likely than other patients to experience problems with quality and safety in their care, suggesting that efforts targeting efficiency and quality among this population might have significant payoffs for health systems. Better understanding of similarities and differences in patient characteristics and health care use in different countries may ultimately inform further efforts to improve care for HNHC patients in these health systems.

**Methods:**

We conducted a cross-sectional descriptive study using one year of patient-level data on high-cost patients in seven high-income OECD member countries. Countries were selected based on availability of detailed information (large enough samples of claims, administrative, and survey data of high-cost patients). We studied concentration of spending among high-cost patients, characteristics of high-cost patients, and per capita spending on high-cost patients.

**Findings:**

Cost-concentration of the top 5% of patients varied across countries, from 41% in Japan to 60% in Canada, driven primarily by variation in the top 1% of spenders. In general, high-cost patients were more likely to be female (57.7% on average), had a significant number of multi-morbidities (up to on average 10 major diagnostic categories (ICD chapters), and had a lower socioeconomic status. Characteristics of high-cost patients varied as well: median age ranged from 62 in the Netherlands to 75 in Germany and the difference in socioeconomic status is particularly stark in the US. Lastly, utilization, particularly for inpatient care, varied with an average number of inpatient days ranging from 6.6 nights (US) to 97.7 nights in Japan.

**Interpretation:**

In this descriptive study, there is substantial variation in the cost concentration, characteristics, and per capita spending on high-cost patient populations across high-income countries. Differences in the way that health systems are structured likely explains some of this variation, which suggests the potential of cross-system learning opportunities. Our findings highlight the need for further studies including comparable performance metrics and institutional analysis.

## Introduction

Health care spending is concentrated among a relatively small number of high-cost patients [1]. These patients face the most pressing medical needs and typically suffer poor clinical outcomes [2–4]. It has been suggested that improved care for this population could increase efficiency and improve overall quality within health systems [5].

Prior studies found that high need, high cost (HNHC) patients tend to be older and tend to suffer from more morbidities than the general population [6–9]. However, we do not know how generalizable this phenomenon is across health systems and what the impact of a health system may be on characteristics and health care use. To date, no study has systematically described HNHC patients across different countries, using patient-level data inclusive of all age groups. A cross-country descriptive comparison of HNHC patients could help us to move beyond assumptions and identify similarities as well as differences in patterns that may inform further efforts to improve care for HNHC patients.

Therefore, we sought to describe the top 5% of patients with the highest medical spending in a given year in seven countries: Canada, England, Germany, Japan, the Netherlands, Spain and the United States. Our research questions were: What are the demographic and clinical characteristics of the top 5% highest spending patients? What is the concentration of spending in top-1% and top-5% high-cost patients? And, what are the differences in healthcare utilization patterns across these countries?

The goal of this research is to better understand the characteristics and healthcare utilization of high-cost populations indifferent health system to facilitate the identification of priorities for future research and action. In addition, this work provides a preliminary exploration of the impact of differing health systems on HNHC and the care they receive.

## Study data and methods

### Ethical committees

This research involved anonymised records and data sets available for analysis. In each country, appropriate permissions have already been obtained from data holders, and it was not possible to identify individuals from the information provided. Therefore, this study was considered exempt from ethical approval. With the exception of Japan, where this study was approved by the Ethics Committee of Kyoto University Graduate School and Faculty of Medicine, Japan (R0438-1) and Aragon, Spain where the study was approved by the regional Ethics Committee of Aragon (PI17/0411).

### Study design, data sources and study sample

We conducted a retrospective observational study of the top 5% high-cost patients in one year in seven high-income countries, Canada, England, Germany, Japan, the Netherlands, Spain, and the United States. Country partners were recruited based on high-quality sources of patient level activity based administrative claims data, survey data, electronic medical records and/or available registry data. Our primary concern with respect to data was ensuring adequate sample sizes to permit reasonable inference about the use of individuals’ medical services. We ensured that the samples included all medical care received by beneficiaries across all age groups, and in the US across all payers.

We used a variety of rich data sources for our analysis. In Canada and Spain, the dataset included all claims from all inhabitants in one region, Ontario and Aragon, respectively. In Germany, data were obtained from two statutory health insurers, whose population is generally more rural and older than the national average. In the Netherlands, high-cost analyses were conducted on all beneficiaries of one insurance company, offering a sample of 20% of the population. Japanese data came from two prefectures of insurance claims of those not currently employed (e.g. farmers, the self-employed, retired, unemployed and their families). (Note: we address concerns of generalizability of this data in the Appendix, see S1 File). In England, we used a database from selected primary care practices (Clinical Practice Research Datalink) that is representative for the population and enriched this information with standardized prices. For the U.S., we relied on utilization and payment data from the Medical Expenditure Panel Survey (MEPS). This is a is a set of large-scale surveys of families and individuals, their medical providers, and employers across the United States. MEPS is considered the most complete source of data on health care utilization in the U.S. and is designed to be representative of the non-institutionalized, community-dwelling population.

The appendix contains a more detailed description of the data sources as well as information on the health care systems and demographics for each country (see S1 File).

We first identified individuals falling into the top 5% (the so-called ‘high-cost’ population) of medical care spending in each country. We did not exclude those individuals with no medical spending in a given year. Activities were costed in each country using a standard approach, generally price times volume, and if pertinent, standardized for between payer and between provider price differences (See S1 File, paragraph 2.7.2).

Costs were categorized according to the International Classification for Health Accounts (ICHA-HC) categories for Inpatient Curative Care (HC1.1); Day and Outpatient Care (HC1.2 and HC1.3, this includes primary care); Rehabilitative and Post-Acute Care (HC2); Outpatient pharmaceuticals (HC5.1.1 and HC5.1.2); and, Other Medical Goods and Ancillary Services (HC5.1.3, HC5.2 and HC4) [10]. A total of 43 country-specific categories of spending were mapped to these ICHA categories. While this substantially coarsens the data, it is a necessary simplification to permit cross-country comparison. Additionally, this mapping was straightforward in most cases. For instance, all pharmaceuticals and drugs were categorized into a single category, the use of primary care and specialists was categorized as outpatient, and the use of imaging and lab tests were categorized as “other.” When uncertainties remained, we reviewed detailed documentation describing expense categories and obtained additional specific descriptions for the items included. The appendix summarizes the costing method and classification schemes for each sample (See S1 File). To permit between-country comparisons, measures of spending were adjusted for purchasing power parity (PPP) to account for differences in cost-of-living and purchasing power between countries [11].

Clinical conditions were described using International Classification of Diseases, tenth revision (ICD-10) codes in all countries except Spain and the U.S., where comparable clinical classification codes (CCC) were used. Prescription drug utilization was classified using the Anatomical Therapeutic Chemical (ATC) Classification System categories, level 2, except for Japan where no official mapping scheme to the ATC was available, and England where equivalent British National Formulary sections were used.

### Analyses

We performed descriptive analyses of demographic and clinical characteristics of patients, and spending and utilization patterns across countries.

We studied relative spending among the high-cost patients between the seven countries according to the ICHA-HC spending categories and our definition of mental health spending (see above).

Because we expected inpatient spending to account for the majority of overall spending, we also examined differences in inpatient admissions and average length of stay.

## Study results

### Patient characteristics

**Fig 1:**
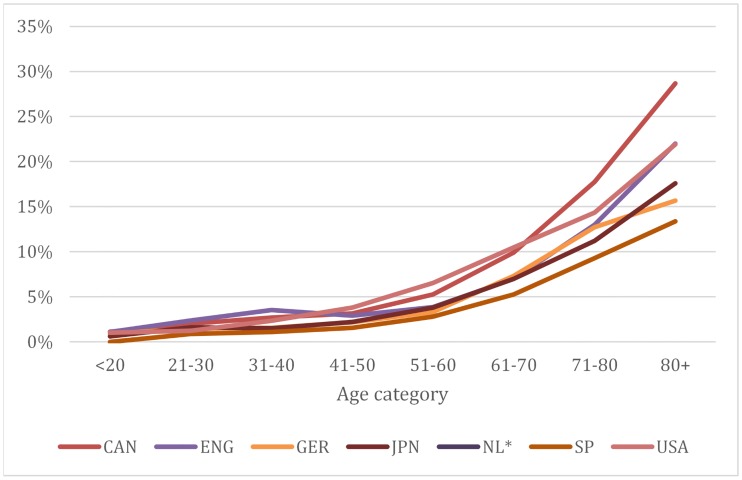
Proportion of high-cost patients as percentage of total population. Notes: Authors’ analysis of administrative or survey data for each region. The Appendix provides a full description of country- or province-specific data sources (see S1 File). Abbreviated countries are Canada, England, Germany, Japan, the Netherlands, Spain and United States. We do not have information for the Netherlands.

In all samples, HNHC patients are more likely to be female and have lower socioeconomic status. The difference in socioeconomic status is particularly stark in the US (Table 1). The median age of HNHC patients varies from 62 (Netherlands) to 75 in Germany; and 40% to 68% of HNHC patients are younger than 70. When we categorized HNHC patients into age groups, we found that older patients in Germany faced a smaller chance of being HNHC than patients in the US, Canada, and England (Fig 1). The mortality rate of HNHC patients during the study period ranged from 7% in the Netherlands to 11% in England. Use of prescription drugs ranged from 8 to 10 distinct categories (excluding England and Japan because of difference in classification methodology). As might be expected, HNHC patients had significant comorbidities, with patients in Japan having conditions across nearly 10 unique ICD chapters (Table 1). The conditions most reported were diseases of the circulatory system (ICD chapter I00-I99), neoplasms (ICD chapter C00-D49) and musculoskeletal illnesses (ICD chapter M00-M99).

**Table 1:** Demographic and clinical characteristics of high-cost patients.

	CAN		ENG		GER	JPN	NL		SP		USA	
Region	Ontario				Kinzigtal				Aragon			
**Demographics (top 5%)**												
% Female		55%		57%	54%	49%		54%		48.90%		56%
Mean age		61		64	68	67		57		67		60
Median age		65		69	75	69		62		71		63
Proportion of patients < 70 years		62%		53%	40%	54%		68%		48.90%		65%
Population mortality rate		8.4%		11.3%	10.8%	9.0%		6.9%		10.3%		NA
Mortality rate > 65		14.0%		NA	14.7%	12.6%		11.7%		13.9%		NA
Socio-economic status (1^st^ is lowest)	1st	22.6%	1st	22.5%	NA	NA	1st	39.0%	1st	0.1%	1st	33.4%
	2nd	20.4%	2nd	21.1%			2nd	34.8%	2nd	77.7%	2nd	21.0%
	3rd	19.3%	3rd	20.2%			3rd	26.2%	3rd	22.1%	3rd	17.0%
	4th	19.2%	4th	19.1%					4th	0.2%	4th	15.7%
	5th	17.9%	5th	17.2%							5th	12.9%
**Clinical characteristics (top 5%)**												
# unique ICD chapters (SD)		6.5 (2.4)		4.1 (2.5)	8.0 (2.9)	9.6 (3.0)		2.5 (1.7)		4.3 (2.4)		5.8 (2.1)
# unique drug codes (SD)		10.0 (4.0)		4.6 (2.1)	9.7 (4.6)	22.6 (11.5)		8.0 (4.8)		8.8 (4.0)		7.8 (4.0)
**Spending**												
Mean Total Cost top 5%		$22,588		$13,078	$27,560	$42,170		$28,508		$14,328		$40,825
Mean Total Cost top 1%		$61,244		$27,297	$61,855	$78,019		$65,766		$29,526		$79,842

Authors’ analysis of data from administrative claims data from the region of Ontario in Canada (CAN, FY 2012–2013), the Kinzigtal region in Germany (GER, 2013), two prefectures in Japan (JPN, FY 2014), a national insurer in the Netherlands (NL, 2012), the region of Aragon in Spain (SP, 2015). Data for the United States came from the Medical Expenditure Panel Survey (US, 2013–2014), and English data from a utilization database from selected primary care practices (Clinical Practice Research Datalink), linked with information from Hospital Episode Statistics and enriched with cost data. The Appendix (see S1 File) provides a full description of country- or province-specific data sources. Socio-economic status is ranked from low (1^st^) to high and based on household individual income level in CAN and US; a compound score per postal code region based on income and education in NL; compound score (index of multiple deprivation) per small geographical area (approximately 1,500 residents) in ENG. Results are presented based on quintiles of population in CAN, ENG and US, and tertials in NL. In Spain, a socioeconomic proxy was based on level of drugs copayment, which in turn is based on household income. 1: no copayment: social salary—long-term unemployment and dole exhausted; 2: <18,000€ annual income; 3:18,000 to 100,000€ annual income; 4: more than 100,000. GER and JPN did not have access to this information. Unique chapters International Classification of Diseases 10th revision: chapter R (symptoms and signs) and Z (other) are excluded; In the Netherlands, claims data for primary care don’t include ICD. International drugs classification using the Anatomical Therapeutical Chemical Classification scheme, level 2 (93 categories), except for Japan and England. In Japan, the National Health Insurance Drug List was used (first 3 digits, 142 classifications), In England equivalent BNF sections were used for primary care prescriptions only. Total spending is based on all medical care. Long-term care and custodial services are excluded. Results are reported in purchasing power parity (PPP)-adjusted USD, adjusting for exchange rate instead of PPP gives similar results. The English dataset did not have the following components available: community care, mental health and some other secondary and primary care and so underestimate total cost by approximately 35%.

### Cost concentration and spending per patient

**Fig 2:**
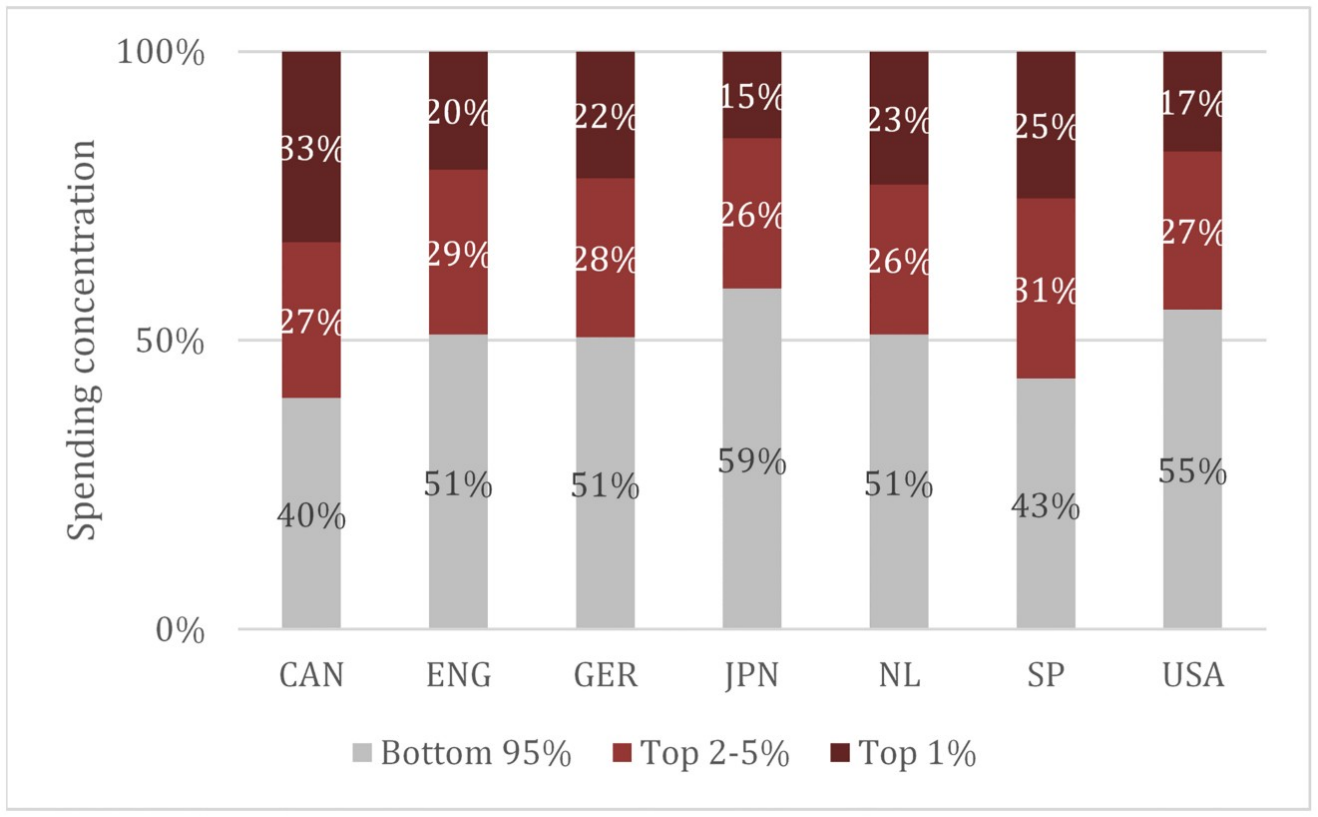
Proportion of medical spending by percentage of population. **Notes**: Authors’ analysis of administrative or survey data for each region. The Appendix provides a full description of country- or province-specific data sources (see S1 File). Abbreviated countries are Canada, England, Germany, Japan, the Netherlands, Spain and United States. Analyses are based on all medical care. Long-term care and custodial services are excluded.

Across all countries, medical spending is highly concentrated, although the exact extent of concentration varies (Fig 2). For instance, in Japan the top 5% of spenders account for 41% of spending, compared to 60% in Canada. These differences appear to be primarily explained by variation in spending among the top 1%, which ranges from 15% in Japan to 33% in Canada. (Variation in spending among the top 2–5% is much smaller, ranging from 26% to 31%).

### Spending and utilization patterns

**Fig 3:**
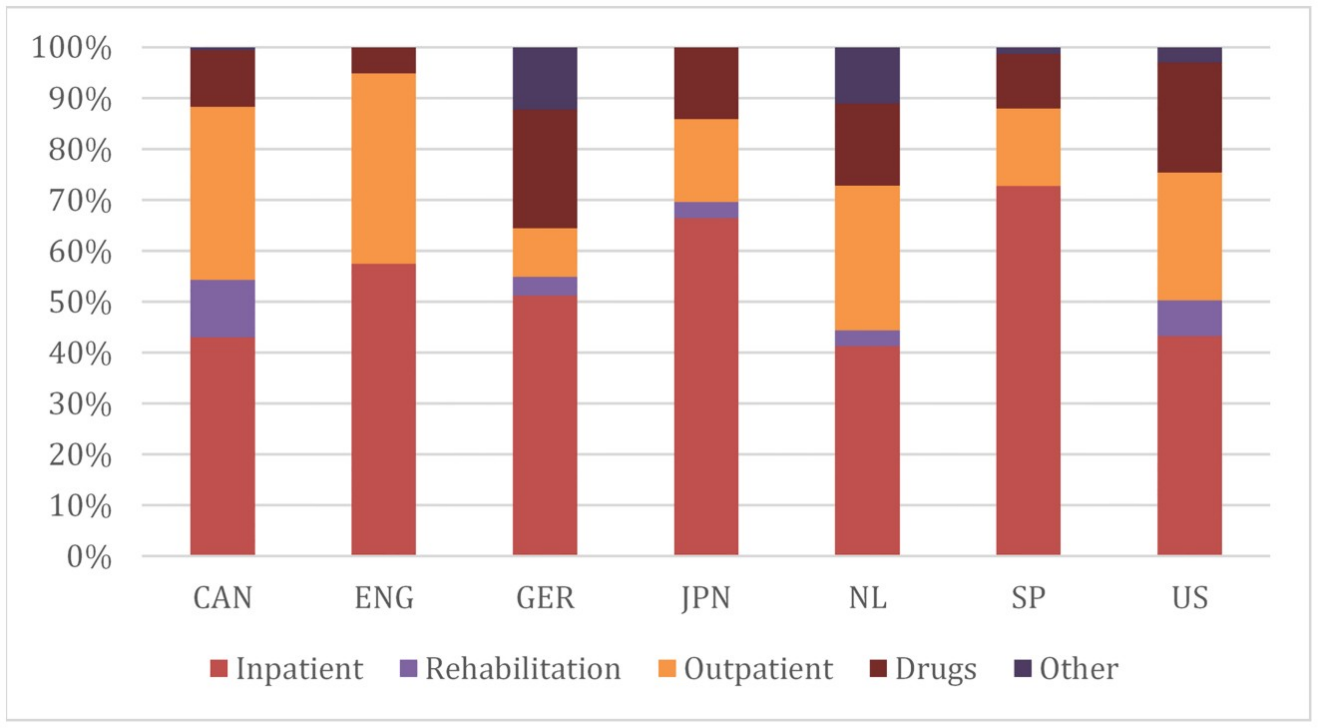
Distribution of medical spending. **Notes**: Authors’ analysis of administrative or survey data for each region. The Appendix provides a full description of country- or province-specific data sources (see S1 File). Abbreviated countries are Canada, England, Germany, Japan, the Netherlands, Spain and United States. Outpatient spending refers to primary care, outpatient specialist care and day curative care except in Germany, where outpatient surgeries and day care is included in the inpatient category. Rehabilitation refers to all rehabilitative care, both inpatient and outpatient (home health). Missing data; ENG, rehab, other; JPN, other; SP, rehab.

Inpatient hospital care is consistently the largest spending category across countries (Fig 3), ranging from 41% of spending in the Netherlands to 73% in Spain. The share of outpatient and office-based hospital care also varies, ranging from 8% in Spain to 37% in England. Similarly, we observe variation in pharmaceutical spending, which accounts for 11% of spending in Spain and Canada to 23% of spending in Germany. The proportion of spending on rehabilitation and post-acute care is largest in the US and Canada, and highest among the elderly populations (> 80 years old).

**Fig 4:**
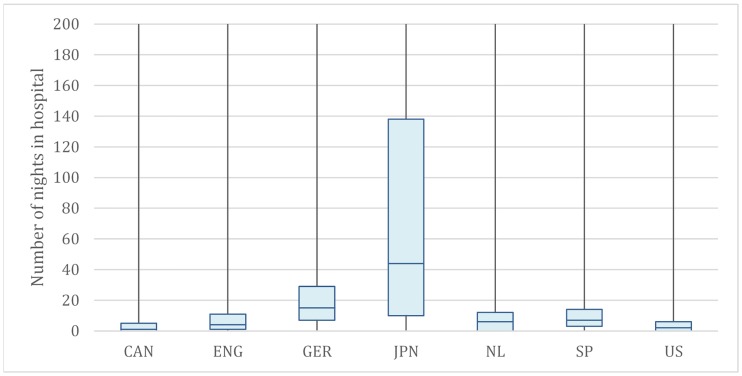
Utilization: Inpatient admissions to hospital. **Notes**: SOURCE Authors’ analysis of administrative or survey data for each region. The Appendix provides a full description of country- or province-specific data sources (see S1 File). Abbreviated countries are Canada, England, Germany, Japan, the Netherlands, Spain and United States. The average number of inpatient days for top 5% high cost population. Inpatient days are defined as the number of nights a patient stays in the hospital. For Canada: *Including both DAD & OMHRS visits.

While inpatient spending consistently accounts for a plurality of spending across countries, we observed substantial variation in use of inpatient services. For instance, the average number of inpatient stays ranged from 6.6 nights (U.S.) to 97.7 nights in Japan. And while 96% of Spanish high-cost patients spent at least 1 night in hospital, only 69% of US HNHC patients do. (Fig 4) As with overall per capita spending, the US remains the most expensive on the inpatient front—the average cost of an inpatient night (PPP-adjusted USD), ranges from $432 in Japan to $3,180 in the U.S.

## Discussion and implications

### Discussion

This study represents the first systematic study describing high-cost patients using high-quality, patient-level data in large samples across different countries. We find that these patients are generally older, more often female, and struggle with several (chronic) conditions. HNHC patients suffer most frequently from cardiovascular diseases, neoplasms and musculoskeletal disorders. Characteristics of these patients vary across countries in terms of absolute spending, hospital utilization patterns, and the distribution of spending by category.

Our findings document some, but not substantial variation in concentration among the top 5% of spenders. Additionally, this variation in cost concentration is almost entirely driven by the top 1% high cost patients. There may be several explanations: First, this group is more likely to use more specific resource-intensive treatments such as very expensive technologies [6]. Indeed, prior research has shown that variation in spending appears to be largely driven by variation in the use of medical technology and health care prices [12], and that the highest prices for labor and goods are found in the U.S. [13,14]. Our findings on the costs of an inpatient day seem to support this: the relatively low rate of hospitalizations, compared with high per unit costs in the U.S. may suggest that further reductions in volume may be less feasible, and that price is a more logical target for interventions.

### Limitations

Our results come with some caveats. Although we undertook substantial efforts to achieve a fair degree of data comparability between countries, the restrictions in data sharing within and across countries, impeded the estimation of proper risk-adjusted measures of HNHC patients' utilization, so the exercise should be confined to a thorough description of the utilization and associated costs for groups of fragile patients in across health systems, not a full comparison of health systems performance. A resulting limitation of this approach was that our analyses were limited to those indicators with highest comparability [15] and that more detailed information within utilization categories, such as the use of diagnostic tests or emergency care. Also, we were not able to assess the impact of medical conditions or the use of specific pharmaceuticals.

Additionally, as costs computing methodologies varied between countries (eg, full costing methods, imputation, estimations based on public tariffs) the overall costs might not be directly comparable. Moreover, most of the regions only include third-party financing, so private payments and out-of-pocket costs are generally not considered. Although in most of our sample of countries these payments are thought to amount for only a small proportion of medical spending in this type of patients, it is uncertain the actual effect.

To see whether our overall expenditure estimations were affected by these limitations, we ranked each country overall expenditure per-capita in HNHC and compared with OECD spending rankings. Notably we got the same ranking for all countries except Japan where just two prefectures were included in the sample. All in all, having different cost-allocation methods has very likely limited the interpretation of differences across spending categories.

On a different line, because our analyses contain one full year of spending, we are able to give a complete overview of high-cost users in any given year, but further analysis is needed to analyse the differences between persistent high-cost users and incidental high-cost users.

Lastly, although sufficiently large, the samples used in our study might not be representative of the universe of HNHC patients then limiting the generalizability of our findings.

### Implications and conclusion

Given that a small share of the population accounts for a substantial plurality of medical spending, improving outcomes and reducing costs requires strategies that focus on these patients. To do so, analyses like ours are prerequisites.

We show that these patients, regardless of health system, suffer from multiple co-morbidities. Prior research suggests that these patients face gaps in care [16], and that lack of care coordination increases health care utilization [17–19]. While it is an open question to what extent better coordination can reduce spending broadly [20], evidence suggests that targeted care coordination programs do increase quality and may reduce costs for specific populations [21–23]. This suggests that better coordination of care before, during and after an inpatient admission may be beneficial.

The heterogeneity of populations within the HNHC group hampers the development of evidence-based strategies to improve conditions for these patients and increase system efficiency. On the other hand, because the patient characteristics of the high-cost patient group appears to be more similar than different across countries may suggest that the differences can be explained by the differences in the organization of different health care systems. Thus, more research focusing on identifying homogenous patient groups within the top 5% highest spenders and study their care pathways may yield many opportunities to identify promising ways for improvements. Additionally, cross-country comparisons also can help because they create larger populations solving possible small-n diseases.

In conclusion, this is the first study, and based on patient level data, which systematically describes HNHC patients across countries. Our findings and the limitations highlight the need for sounder cross-national comparisons of high-cost, high-need patients using prospectively designed quality and cost metrics, including more specific information on (combinations of) medical conditions and health care use, institutional analysis, and risk-adjustment or stratification of individuals. Further work along these lines hold strong promise to improve patients’ lives as well as systems’ performance. Ultimately, the results of this project provide input for countries to prioritize policy targets to improve care for their high-cost population.

## Transparency & author statement

The lead author (the manuscript’s guarantor) affirms that the manuscript is an honest, accurate, and transparent account of the study being reported; no important aspects of the study have been omitted; and any discrepancies from the study as originally planned (and, if relevant, registered) have been explained.

All authors agree to be accountable for all aspects of the work and approved the final manuscript for submission.

## Supporting information

S1 File Appendix to ‘A challenge to all. A primer on inter-country differences of high-need, high-cost patients’. (DOCX)

## References

[1] French E, Kelly E. Medical Spending around the Developed World. Fisc Stud. 2016;37(3–4):327–44. 10.1111/j.1475-5890.2016.12106 PMC6680320 31404348

[2] Alexandre LM. High-cost patients in a fee-for-service medical plan. The case for earlier intervention. Med Care. 1990;28(2):112–23. 2105413 10.1097/00005650-199002000-00002

[3] Coughlin TA, Long SK. Health care spending and service use among high-cost medicaid beneficiaries, 2002–2004. Inquiry. 2009;46(4):405–17. 20184167 10.5034/inquiryjrnl_46.4.405

[4] Aleckxih L, Shen S, Chan I, Taylor D, Drabek J. Individuals Living in the Community with Chronic Conditions and Functional Limitations: A Closer Look. Lewin Group. Washington D.C.; 2010.

[5] Blumenthal D, Chernof B, Fulmer T, Lumpkin J, Selberg J. Caring for High-Need, High-Cost Patients—An Urgent Priority. NEJM. 2016; 10.1056/NEJMp1608511 27602661

[6] Wammes JJG, Tanke MA, Jonkers W, Westert GP, Van der Wees P, Jeurissen PP. Characteristics and healthcare utilisation patterns of high-cost beneficiaries in the Netherlands: a cross-sectional claims database study. BMJ Open. 2017;7(11):e017775 10.1136/bmjopen-2017-017775 29133323 PMC5695517

[7] Wodchis WP, Austin PC, Henry DA. A 3-year study of high-cost users of health care. CMAJ. 2016;188(3):182–8. 10.1503/cmaj.150064 26755672 PMC4754179

[8] Calver J, Brameld KJ, Preen DB, Alexia SJ, Boldy DP, McCaul KA. High-cost users of hospital beds in Western Australia: a population-based record linkage study. Med J Aust. 2006 4 17;184(8):393–7. 16618238 10.5694/j.1326-5377.2006.tb00289.x

[9] Riley GF. Long-term trends in the concentration of medicare spending. Health Aff. 2007;26(3):808–16. 10.1377/hlthaff.26.3.808 17485760

[10] OECD, Eurostat, WHO. A System of Health Accounts. 2011.

[11] Organization for Economic Co-operation and Development. Purchasing power parities (PPP). 2017 [cited 2017 Jun 12]. https://data.oecd.org/conversion/purchasing-power-parities-ppp.htm

[12] Papanicolas I, Woskie LR, Jha AK. Health Care Spending in the United States and Other High-Income Countries. JAMA. 2018 3 13;319(10):1024 10.1001/jama.2018.1150 29536101

[13] Dieleman JL, Squires E, Bui AL, Campbell M, Chapin A, Hamavid H, et al Factors Associated With Increases in US Health Care Spending, 1996–2013. JAMA. 2017;318(17):949–54. 10.1001/jama.2017.15927 PMC5818797 29114831

[14] Papanicolas I, Jha AK. Challenges in International Comparison of Health Care Systems. JAMA. 2017 7 27;148(8):956–7. 10.1001/jama.2017.9392 28750110

[15] Schreyögg J, Stargardt T, Velasco-Garrido M, Busse R. Defining the “health benefit basket” in nine European countries: Evidence from the European Union Health BASKET Project. Eur J Heal Econ. 2005;6(SUPPL. 1):2–10. 10.1007/s10198-005-0312-3 PMC1388078 16270212

[16] Schoen C, Osborn R, Squires D, Doty M, Pierson R, Applebaum S. New 2011 Survey of Patients With Complex Care Needs in Eleven Countries. Health Aff. 2011;30(12):2437–48. 10.1377/hlthaff.2011.0923 22072063

[17] Druss BG, Marcus SC, Olfson M, Tanielian T, Elinson L, Pincus HA. Comparing the national economic burden of five chronic conditions. Heal Aff. 20(6):233–41. 10.1377/hlthaff.20.6.233 11816664

[18] Nyweide DJ, Anthony DL, Bynum JPW, Strawderman RL, Weeks WB, Casalino LP, et al Continuity of care and the risk of preventable hospitalization in older adults. JAMA Intern Med. 2013;173(20):1879–85. 10.1001/jamainternmed.2013.10059 24043127 PMC3877937

[19] Hansen J, Groenewegen PP, Boerma WGW, Kringos DS. Living in a country with a strong primary care system is beneficial to people with chronic conditions. Health Aff. 2015;34(9):1531–7. 10.1377/hlthaff.2015.0582 26355055

[20] McWilliams JM. Cost Containment and the Tale of Care Coordination. N Engl J Med. 2016;375(23):2218–20. 10.1056/NEJMp1610821 27959672 PMC5506340

[21] Blumenthal D, Abrams MK. Tailoring Complex Care Management for High-Need, High-Cost Patients. JAMA—J Am Med Assoc. 2016;316(16):1657–8. 10.1001/jama.2016.12388 27669168

[22] Hsu J, Vogeli C, Price M, Brand R, Chernew Michael E Mohta N, Chaguturu SK, et al Substantial Physician Turnover And Beneficiary “Churn” In A Large Medicare Pioneer ACO. Health Aff. 2017;36(4):640–8. 10.1377/hlthaff.2016.1107 28373329

[23] Melnick GA, Green L, Rich J. House Calls: California Program For Homebound Patients Reduces Monthly Spending, Delivers Meaningful Care. 2016;35(1):28–35. 10.1377/hlthaff.2015.0253 26733698

