# Supplementary material for: A challenge to all. A primer on inter-country differences of high-need, high-cost patients
Source: PLoS One. 2019 Jun 19;14(6):e0217353. doi: 10.1371/journal.pone.0217353 (PMC6583982; doi:10.1371/journal.pone.0217353)
Supplement: S1 File — (DOCX) [file pone.0217353.s001.docx]

A challenge to all. A primer on inter-country differences of high-need, high-cost patients.

Appendix

# Health Care Systems and Data Sources

## Health care systems

To assess the impact of a range of health care delivery systems, we analyzed data from nations with widely varying health systems.

**Payers:** The United States has a multipayer health care system, with a mix of public (over 65 years old) and private (under 65) payers. Germany has a compulsory, multi-payer social insurance systems, like the Netherlands, however, the Netherlands are transitioning to a market-based payment system. Also, in Japan, medical care is paid for by mandatory universal insurance. This is provided through employment or through the local government. Insurance is financed by both premiums and government subsidies. Additional private insurance can be purchased. Canada and Spain have a single-payer public insurance system per province / region. England has a public National Health System.

**Delivery system:** Providers are largely private in the USA, Netherlands, Canada, and Germany. The USA has a mix of profit and not-for profit providers, in the other countries, providers are mainly not-for-profit. In Japan, the majority of hospitals and clinics are private and non-profit; remainder are public, with larger hospitals tending to be public. In Spain primary care and hospital providers are mainly public, although dental care or pharmaceutical is mainly provided by the private sector. In England while care is free at the point of use (apart from some dental care) providers are a mix of public and private providers, notably primary care is largely provided through a network of general practitioners who are private providers under contract to the National Health Service.

#### eTable 1: Health Care System and Population Demographics

|  | **Canada** | **England** | **Germany** | **Japan** | **Netherlands** | **Spain** | **US** |
| --- | --- | --- | --- | --- | --- | --- | --- |
| **HEALTH SYSTEM** |  |  |  |  |  |  |  |
| **General features*** | Regionally administered universal public insurance program | National Health System | Statutory health insurance system | Statutory health insurance system | Statutory health insurance system | Single public payer | Mix of private and public (Federal and state-level) |
|  |  |  |  |  |  |  |  |
| **Insurance model*** | Single public payer by province | Single public payer (NHS) | Competing SHI insurers; high income can opt out for private coverage | Regional system with public insurance | Universally mandated private insurance (national exchange); | Mandatory Universal coverage. | Multipayer insurance. Coverage mandated. 10.4% of adults uninsured) |
| **% Population insured*** | 100 | N/A | 100 | 100 | 99 | Close to 100 | 90 |
| **Reimbursement structure medical care*** | Global, lump-sum payments | Global, lump-sum payments combined with per-patient payments by DRG | Payment for outpatient physicians is fee-for-service with a budget cap, hospital payment is DRG. | DRG-like per-patient payments | Population-based payment for primary care; Otherwise: DRG-like per-patient payments | Hospital: global, lump-sum payment, plus negligible episode-payment based on DRG; Primary care: global, annual lump-sum | Mainly per patient payment per DRG, alternative population-based contracts |
| **Health care spending per capita (USD PPP)**** | 4,351 | 3,235 | 4,819 | 3,713 | 5,131 | 2,898 | 8,713 |
| **Health Care Spending %GDP**** | 10.2 | 8.5% | 11 | 10.2 | 11.1 | 8.8 | 16.4 |
|  |  |  |  |  |  |  |  |
| **DEMOGRAPHICS** |  |  |  |  |  |  |  |
| **Median age (years)** | 42.2 | 40 | 46.3 | 46.5 | 42.2 | 43 | 37.7 |
| **Population aged 65 and older (%)**** | 14 | 19 | 21 | 23 | 15 | 17 | 13 |
| **Life expectancy at birth (yrs)**** | 81.5 | 83.2 | 80.9 | 83.4 | 81.4 | 83.2 | 78.8 |

*‘* Data come from Commonwealth Fund International Profile (2015)**^[[1]](#endnote-1)^; *Data come from OECD Health at a Glance (2015); Additional country-specific information from WHO health system reviews.*^[[2]](#endnote-2)^

On the delivery side, Canada, Spain, England and the Netherland have a strong focus on primary care, with General Practitioners (GPs) coordinating the care of patients and acting as gatekeepers for access to more specialist care.

**Benefit package:** The benefit package inevitably affects which patients are in the top 5% highest spending cohort per country. Previous studies have reflected on the differences in benefit package and covered services ^[[3]](#endnote-3),^^[[4]](#endnote-4),^^[[5]](#endnote-5),^^[[6]](#endnote-6)^. All countries in our study, cover regular medical services, and exclude cosmetic surgery. Previous studies only found small differences between the countries. Examples of such differences are related to specific services such as dental care, physical therapy, and prescription drugs. Information on dental care is not available in most countries. In the Netherlands, physical therapy for adults is partially covered (after 20 treatment sessions) for certain chronic conditions. In England, physical therapy is fully covered, while Germany charges co-payments for physical therapy treatments. A potentially bigger difference might be the range of services that is covered within medical care as opposed to long-term care and social services. The countries differ widely on the spending on long-term care and social services^[[7]](#endnote-7)^. That means that limited coverage for health services in *medical care* for health services may be related to potential coverage outside the medical care system. For example, in the Netherlands, short-term home health and physical therapy services for residents in nursing homes are covered through are payed for by the long-term care budget. ^5^

**Reimbursement structure**: In the USA payers negotiate tariffs with providers. The majority of reimbursements are based on Fee-For-Service (FFS) schemes, however there is a transition towards alternative contracts, such as shared savings contracts and capitation. In the Netherlands, hospitals (in- and outpatient care) and rehabilitation centers are mainly reimbursed via payment products similar to Diagnosis Related Group (DRGs). Primary care providers are payed based on a combination of capitation and pay for performance. Spain works with devolved regional budgets that translate into annual lump-sum budgets for hospital and primary care providers, with a small capitation-based payment for primary care physicians. In England, Healthcare Resource Groups (HRGs) are the currency used to set national prices for acute inpatient services. Set mandatory national prices are used for most acute inpatient services. For those services which do not have national prices, reimbursement is determined by local negotiation between provider and commissioner. Reimbursement for primary care services is similarly negotiated between key purchasers’ and providers’ associations at central level. There are also set fees available to GPs for delivering some services, such as flu vaccinations.

## Data sources

Data availability statement: Primary relevant data are within the manuscript and its Supporting Information files. The access to data supporting the manuscript findings is restricted in accordance with data sharing regulations within and across countries. These policies require that access to data be limited to persons who require such access to perform their role on an approved Project or Third-Party Project. As a consequence, truncated aggregated data may be accessed upon request, contacting the author of the manuscript.

#### eTable 2: Data source coverage

|  | **Canada** | **England** | **Germany** | **Japan** | **Netherlands** | **Spain** | **US** |
| --- | --- | --- | --- | --- | --- | --- | --- |
| **Type of data** | Claims data | Linked national health service data | Claims data | Claims data | Claims data, | Linked regional health service data lake | MEPS, self-reported utilization + actual payments |
| **Source population** | Province of Ontario | England | Two statutory health insurers in Baden-Württemberg | Two prefectures | One insurer operating nationwide | Region Aragon | Sampled population U.S. |
| **Number of lives in sample** | 13.6 million | 300,000 | 30.000, older and more rural | about 1.5 million per year | 3.3 million | 1,3 million | 71,815* |
| **Coverage** | 100% Ontario |  |  |  | 20% of Dutch population | 100% Aragon | Sample is made national representative using weighting factors |
| **# high-cost patients** | 729,870 | 15,000 | 1,518 | 72,365 | 166,000 | 65,179 | 2,842* |
| **Year** | FY2012-2013 | FY 2014-2016 | 2013 | FY2014 | 2012 | 2015 | 2013 / 2014 |
|  |  |  |  |  |  |  |  |

*‘* 71,815 observations, weighted to be representative of the 317 million population. Of these, 2,842 were in the top 5 percent of adjusted spending, representing 15.8 million people in the population*

In Canada and Spain, the dataset included all claims from all inhabitants in one region, respectively Ontario and Aragon. In Germany, data were obtained from two statutory health insurers, whose population is generally more rural and older than the national average. In the Netherlands, high-cost analyses were done on all beneficiaries of one insurance company, resulting in a representative sample of 20% of the population. Japanese data came from two prefectures of insurance claims of population of not working in companies (e.g. farmers, the self-employed, retired, unemployed and their families). England, with its National Health System, doesn’t have claims data in the same way as the other countries. Here, we started with a utilization database from selected primary care practices (Clinical Practice Research Datalink) representative for the population and enriched this information with standardized prices. Likewise, in the fragmented US health system, no single administrative data would allow us to analyze a representative sample of the population. Therefore, we used utilization and payment data from the Medical Expenditure Panel Survey (https://meps.ahrq.gov/mepsweb/). This is a is a set of large-scale surveys of families and individuals, their medical providers, and employers across the United States. Health Care is based on insurance data and spending is based on actual payments. MEPS is the most complete source of data on the cost and use of health care in the USA and constructed in a way that it is representative for the non-institutionalized, community-based US population. This appendix contains a detailed overview per country.

## Methodology

#### eTable 3: Overview of ICHA-HC functions included in data source per country

| **ICHA-HC category** | **Canada** | **England** | **Germany** | **Japan** | **NL** | **Spain** | **USA** |
| --- | --- | --- | --- | --- | --- | --- | --- |
| **HC.1 Curative care** |  |  |  |  |  |  |  |
| *HC.1.1 Inpatient curative care* | x | x | x | x | x | X | x |
| *HC.1.2 Day curative care* | x | x | x | x | x | X | x |
| *HC.1.3 Outpatient curative care* | x | x | x | x |  | X | x |
| *Specific: Dental care* | - | - | - | - | P | - | x |
| *HC.1.4 Home-based curative care* | x | x | x | x | x | X | x |
| **HC.2 Rehabilitative care** |  |  |  |  |  |  |  |
| *HC.2.1 Inpatient rehabilitative care* | x | x | x | x | x | X | x |
| *HC.2.2 Day rehabilitative care* | - | x | x | x | x | X | x |
| *HC.2.3 Outpatient rehabilitative care* | - | x | x | x | x | x | x |
| *HC.2.4 Home-based rehabilitative care* | x | - | x | x | P | - | x |
| **HC.4 Ancillary services** |  |  |  |  |  |  |  |
| *HC.4.1 Laboratory services* | x | x | x | x | x | P | x |
| *HC.4.2 Imaging services* | x | x | x | x | x | P | x |
| *HC.4.3 Patient transportation* | - | x | x | - | x | - | x |
| **HC.5 Medical goods (non-specified by function)** |  |  |  |  |  |  |  |
| *HC.5.1 Pharmaceuticals and other medical non-durable goods* | P | x | x | x | x | X | x |
| *HC.5.2 Therapeutic appliances and other medical goods* | P | ? | x | P | P | - | x |

*X = function is included in dataset; - = function is not included in dataset; P = function is partially included in dataset*

### ICHA-HC functions

We used the internationally acknowledged health accounts methodology (ICHA), as described by the OECD and WHO. ^[[8]](#endnote-8)^ *Medical care* was described as the ICHA Health Care Functions: HC 1 Curative Care; HC 2 Rehabilitative Care; HC 4 Ancillary Services; HC 5 Medical Goods; utilization from HC 6 Preventive Care was only included if billed on an individual level. We excluded custodial and long-term care (HC 3) and care delivered at population level (HC 6; HC 7; HC 9).

### Classifications of clinical conditions and drugs categories

Clinical conditions were described using International Classification of Diseases, tenth revision (ICD-10).^[[9]](#endnote-9)^ This classification system breaks down all diseases in 20 categories, of which we excluded chapter R (Symptoms and signs not elsewhere classified) and chapter Z (Other) in our analyses. All countries, except the US, used this classification system. However, there were differences in how and when countries register these conditions. For example, in the Netherlands, clinical conditions based on ICD-codes are only registered for hospital services (in- and outpatient) and only one code per DRG. In other countries, multiple ICD-codes can be captured per visit. Also, both in Canada as the USA, payments can be higher if a patient is sicker. This can be an incentive for more complete registration of co-morbidities. It is not clear whether this bias exists in the MEPS dataset for the USA, however, given that MEPS is not drawn from administrative data and may not account for all coded diagnoses.

Drug use was classified in terms of Anatomical Therapeutic Chemical (ATC) Classification System categories, level 2. Drug use was classified in terms of Anatomical Therapeutic Chemical (ATC) Classification System categories, level 2 (93 categories). The USA, Canada and Japan do not use ATC codes. In the USA and Canada, respectively National Drug Codes (NDC) and Drug Identification Number (DIN) codes were mapped to ATC categories. Japan used the drug code of National Health Insurance Drug List, which consists of 142 categories. England used the British National Formulary classification system, at the section level.

# Per country

## Canada

### Health Care System

The Canadian health care system is a publicly funded system that provides universal coverage for medically necessary health care services provided on the basis of need, rather than the ability to pay. Under Canada’s constitution, the provincial and territorial governments are responsible for establishing, maintaining and managing health care for their residents with financial contributions from the federal government and tax transfers. The federal government is also responsible for delivering services for certain groups of people throughout the country. The five principles of the Canada Health Act include: public administration, Comprehensiveness, universality, accessibility and portability (between provinces).^9^

In Ontario, this translates to all or part of the cost of medical care for residents, including visits to family doctors and specialists as well as most basic and emergency health care services, including hospital stays. Health care is paid for under the Ontario Health Insurance Plan (OHIP) and funded through residential and commercial tax revenue.^[[10]](#endnote-10)^

### Data Source

We obtained data from the Institute for Clinical Evaluative Sciences (ICES) which comprises linked databases of universal coverage health service records for Ontario residents. Data was extracted from the Registered Persons Database (RPDB) from April 1st, 2012 to March 31st, 2013. The RPDB provides basic demographic information (age, sex, location of residence, date of birth, and date of death for deceased individuals) for those issued an Ontario health insurance number. The RPDB also indicates the time periods for which an individual was eligible to receive publicly funded health insurance benefits and the best known postal code for each registrant on July 1st of each year.

The costing methodology, extensively described elsewhere, ^[[11]](#endnote-11)^ uses a two-step approach to allocate healthcare costs to individual patients: 1) identify each individual’s encounters (i.e., utilization) with the healthcare system, and 2) attach unit costs/prices to services utilized during the encounter. Patient encounters are grouped into short episodes, long episodes, and visits/claims (Appendix exhibit 4). The measure of utilization and unit costs used differ based on the type of encounter. Generally, inpatient hospital-based encounters are grouped as short episodes; and their costs are computed by multiplying resource intensity weights (RIW) (i.e., a measure of how much resources were utilized during the encounter) with cost per weighted case (CPWC). CPWC method calculates the cost of a hypothetical, average Ontario patient; not the actual cost of a specific patient. Thus, inpatient hospitalization costs may differ between patients because of the variations in RIWs. For longer term episodes such as complex continuing care, different utilization measures are used and unit costs based on weighted days. For visit/claim-based encounters, these costs are generally determined at the instance of utilization; and are directly recorded in the respective database. The methods used for calculating the utilization weights and unit costs in the short and long episodes were developed by the Canadian Institute for Health Information (CIHI).

A description of the utilization databases are provided below:

Discharge Abstract Database (DAD) – The DAD is compiled by the Canadian Institute for Health Information and contains administrative, clinical (diagnoses and procedures/interventions), demographic, and administrative information for all admissions to acute care hospitals, rehab, chronic, and day surgery institutions in Ontario. At ICES, consecutive DAD records are linked together to form ‘episodes of care’ among the hospitals to which patients have been transferred after their initial admission.

Same- Day-Surgery (SDS) - The SDS is compiled by the Canadian Institute for Health Information and contains administrative, clinical (diagnoses and procedures) and demographic information for all patient visits made to day surgery institutions in Ontario. The main data elements include patient demographics, clinical data (diagnoses, procedures, physician), administrative data (institution/hospital number etc.), financial data, service-specific data elements for day surgery and emergency.

National Ambulatory Care Reporting System (NACRS) - The NACRS is compiled by the Canadian Institute for Health Information and contains administrative, clinical (diagnoses and procedures), demographic, and administrative information for all patient visits made to hospital- and community-based ambulatory care centres (emergency departments, day surgery units, hemodialysis units, and cancer care clinics).

National Rehabilitation Reporting System (NRS) - The NRS is compiled by the Canadian Institute for Health Information and contains client data collected from participating adult inpatient rehabilitation facilities and programs across Canada. Main data elements contain socio-demographic information, administrative data (e.g. referral, admission and discharge), health characteristics, activities and participation (e.g. ADL, communication, social interaction), and interventions.

Ontario Mental Health Reporting System (OMHRS) - The OMHRS is compiled by the Canadian Institute for Health Information and contains administrative, clinical (diagnoses and procedures), demographic, and administrative information for all admissions to adult designated inpatient mental health beds. This includes beds in general hospitals, provincial psychiatric facilities, and specialty psychiatric facilities. Clinical assessment data is ascertained using the Resident Assessment Instrument for Mental Health (RAI-MH), but different amounts of information are collected using this instrument depending on the length of stay in the mental health bed.

Ontario Health Insurance Plan Claims Database (OHIP) - The OHIP claims database contains information on inpatient and outpatient services provided to Ontario residents eligible for the province’s publicly funded health insurance system by fee-for-service health care practitioners (primarily physicians) and “shadow billings” for those paid through non-fee-for-service payment plans. The main data elements include patient and physician identifiers (encrypted), code for service provided, date of service, associated diagnosis, and fee paid.

Home-care Database (HCD) - The HCD is a clinical client-centric database that captures all services that are provided by or coordinated by Community Care Access Centres (CCACs). The data elements captured include information on: client, intake, assessment, admission & discharge, diagnosis and surgical procedure, and care delivery. ICES receives home care data from the Ontario Ministry of Health and Long-Term Care (MOHLTC).

#### eTable 4. Summary of services types, weights and unit cost title

| **Type of Service** | **Utilization Database** | **Utilization Unit** | **Weight Name** | **Unit Cost** |
| --- | --- | --- | --- | --- |
| **Group 1: Short Episodes** (mean<60 days) | | | | |
| Acute Hospitalization | DAD | Weighted Case | RIW | CPWC |
| Same Day Surgery & Outpatient | NACRS | Weighted Case | RIW | CPWC |
| Emergency Department | NACRS | Weighted Case | RIW | CPWC |
| Inpatient Rehabilitation | NRS | Weighted Case | Rehabilitation Cost Weight (RCW) | CPWC |
| **Group 2: Longer Term Episodes** | | | | |
| Complex Continuing Care | CCRS | Weighted day | Case Mix Index (CMI) | Cost per Rug-Weighted Patient Day (CPRWPD) |
| Long Term Care Home | CCRS | Weighted day | CMI | CPRWPD |
| Inpatient Mental Health | OMHRS | Weighted day | SCIPP CMI | CPMHWD |
| **Group 3: Visits/Claims** | | | | |
| Primary and Specialist Physician | OHIP | Visit | n/a | Fee Paid |
| Home Care | OHCAS, HCD | Visit | n/a | Cost/visit |
| Pharmaceuticals | ODB | Prescription | n/a | Amount Paid |
|  |  |  |  |  |

Ontario Drug Benefit Claims (ODB) - The ODB database contains prescription medication claims for those covered under the provincial drug program, mainly: those aged 65 years and older, nursing home residents, patients receiving services under the Ontario Home Care program, those receiving social assistance, and residents eligible for specialized drug programs. Main data elements include drug identifier, quantity, # days supplied, date dispensed, cost, and patient, pharmacy and physician identifiers.

### Study Population

Our overall analytic sample comprised 14,597,407 Ontario residents, eligible for OHIP on July 1, 2012, 729,870 of whom were in the top 5% of spending.

Costs associated with long-term care and personal service/homemaking were excluded from the data.

### Limitations and Adjustments

*Classification of Mental Health patients*

In order to compute mental health costs and classify patients into mental health high cost users, we restricted the existing cost methodology to focus only on mental health care utilization.

From DAD, only main diagnoses from the ‘F’ chapter of ICD-10-CA were included. In NACRS, any visit with an ICD-10-CA diagnosis from the ‘F’ chapter or self-harm subset (X6-X8) was included, as well as acute alliance mental health intervention codes. In OHIP, billings from psychiatrists, ECT procedures and mental health lab tests (e.g lithium tests) were included. From ODB, costs for anti-depressants, antipsychotics, mood stabilizers (valproic acid, lithium) and benzodiazepines were included. Home-care costs were obtained for psychology billings.

Total mental health costs were calculated by adding these sector-specific costs with the OMHRS cost that was calculated using the original costing method. If a patient’s total mental health cost was more than 50% of their total healthcare costs, they were considered a mental health high cost patient.

#### Payment adjustment.

CPWC method calculates the cost of a hypothetical, average Ontario patient having a stay/visit with the given resource intensity, not the actual costs that the specific patient in question incurred. The true CPWC will vary by hospital (teaching vs. non-teaching) and geography (e.g., cost of living variations). CPWC method captures ALL costs for patients regardless of the specific reason for treatment. They are not disease/condition-specific. In contrast to CPWC methods, costs obtained from billings (OHIP, ODB, Home Care) remain accurate in most situations

Resource Intensity Weight (RIW) is not a direct estimate of cost - it is an average across organizations that submit data to the CIHI costing group (20+ in Ontario and 2 in Alberta). Two hospitalizations with similar RIW values could have significantly different actual costs in dollar terms.

The cost methodology draws on 16 different sources of health care utilization and spending, each having its own methods and implications. The cost estimates depend on the way that utilization data is collected.

## England

### Health Care System

The English National Health Service provides universal access to healthcare to the resident population (those ordinarily resident, European union nationals, and other migrants following payment of an NHS surcharge with a visa application) financed from taxes. Non-resident patients have access to some care that is free at the point of use; including general practice consultations, and treatment in general practice, treatment in accident and emergency department, and some infectious disease treatment. For resident patients all primary and secondary care is provided free at the point of use, though most patients are required to co-pay for elective dental care, and prescriptions (based on ability to pay). Healthcare is largely commissioned locally by ‘clinical commissioning groups’ and delivered by a network of public (predominant in in-patient and outpatient hospital care), independent contractors (general practitioners), independent providers (including private providers, voluntary organizations, and social enterprises).^[[12]](#endnote-12)^

Healthcare Resource Groups (HRGs) are the currency used to set national prices for acute inpatient services. Set mandatory national prices are used for most acute inpatient services. For those services which do not have national prices, reimbursement is determined by local negotiation between provider and commissioner. Reimbursement for primary care services is similarly negotiated between key purchasers’ and providers’ associations at central level. There are also set fees available to GPs for delivering some services, such as flu vaccinations.

The majority of long term care, social care and public health is not provided via the national health service. Social care, both residential and non-residential is provided through public and private providers (both for and non-profit).^[[13]](#endnote-13)^ For those in receipt of publicly funded provision, social care is largely commissioned by local government.

### Data source

We obtained data from the Clinical Practice Research Datalink (CPRD) for England. The dataset extraction covered 1^st^ April 2014 – 31^st^ March 2016. The CPRD provides data on care delivered in primary care, including information on consultations (such as face-to-face surgery consultations, telephone consultations and home visits) with General Practitioners (i.e. Family Physicians) and practice nurses. This data was linked to Hospital Episode Statistics (HES) data, including information on attendances at emergency departments, inpatient admissions to hospital, and appointments and procedures at outpatient clinics. These data were enriched with mortality data from the Office for National Statistics (ONS) mortality linkage, and socio-economic deprivation using Index of Multiple Deprivation (IMD) 2015 deciles at the patient ‘small level area’ (LSOA).

### Population

We extracted a sample of patients registered and active with the general practice at 31^st^ March 2014.

We obtained a random sample of patients registered at a general practice participating in Clinical Practice Research Datalink.^[[14]](#endnote-14)^ Our overall analytic sample consisted of 300,000 individuals. Patients in the practices participating in the CPRD are representative of patients registered to practices in England.^[[15]](#endnote-15)^ No restrictions were made on the sample and we did not exclude based on age, prior health care utilization or any other characteristics. Therefore, it is possible for patients in the sample to have no recorded utilization during our study period. The sample included patients who were living in the community and in long-term care.

High-cost patients are defined as the top 5% patients in the sample with highest total costs based on the available data on direct costs to the NHS.

Health care utilization and costs were calculated at an individual patient level across primary and secondary care for a two year period from March 2014 to March 2016. In primary care, we have quantified and attached costs to contacts with clinical staff (GPs and nurses), diagnostic tests, immunizations and prescriptions for medicines and devices.^[[16]](#endnote-16)^ In secondary care, we have quantified utilization in accident and emergency departments, spells of admitted patient care, and attendance at outpatient clinics. The national tariff payment system was used to calculate healthcare resource groups (HRGs) for each occurrence of care and a cost was attached to each HRG. ^[[17]](#endnote-17)^

### Limitations and adjustments

Whilst the CPRD data are representative of patients in England in terms of their clinical and demographic characteristics, the data come from providers of primary care using electronic health record systems. The use of electronic health record systems may vary across the different regions of England; as a result, the data may not be representative at a regional level.

#### ICHA-HC Functions

Data include utilization of primary care (contacts with nurses or general practitioners in general practice, diagnostic tests, immunizations and prescriptions) and secondary care (attendances at emergency departments, inpatient admissions and procedures, and appointments and procedures in an outpatient setting). Data do not include utilization and costs associated with palliative care, prescriptions dispensed in secondary care, and those arising from social care. In addition, there is no information on over the counter drug consumption, or whether written prescriptions were dispensed.

| Category | National database (ICHA-HC functions) |
| --- | --- |
| Inpatient | HC 1.1 Inpatient Curative Care |
| Outpatient | HC 1.2 Day Curative Care and HC 1.3 Outpatient Curative Care |
| Rehabilitation | No costs available |
| Drugs | HC 5.1 Pharmaceuticals. Excludes prescriptions dispensed in secondary care. Inpatient medication is included in HC 1.1 |
| Other | No costs available |

#### Payment adjustment / standardization.

As specific encounters are not attached to specific costs in the NHS (as billings would be in insurance funded systems), determining exact costs arising from each encounter is not possible. For example, General Practices are largely funded via block contracts for a patient list, they are not paid a sum for individual appointments.

Therefore, to cost health care activity for this project, we enumerated all healthcare utilization in the linked dataset, and assigned a cost to each activity. The costs for each activity were taken from a variety of sources, including national reference costs for Healthcare Resource Groups for inpatient admissions, accident and emergency attendances and outpatient attendances ^[[18]](#endnote-18)^, item specific prescription costs linked to a national drug tariff,^[[19]](#endnote-19)^ and estimated reference costs were used for general practice and other activity in primary care. ^[[20]](#endnote-20)^

For the purposes of this project, all admissions recorded in HES were split into either outpatient or inpatient care. Costs associated with day cases or any admission with no overnight stay in HES were classified as outpatient; while costs associated with any admission with an overnight stay were classified as inpatient costs. All emergency department attendances were classified as an outpatient cost. All primary care activity, excluding prescriptions, was also included in the outpatient cost. Prescription costs, only includes primary care prescriptions.

While the figures presented in this report are representative of the underlying dataset, they do underestimate total health care expenditure as a number of components are missing from this data. Examples of missing costs are costs associated with community care, specialized mental health and some primary and secondary care costs, including high cost drugs, not captured in this dataset. Previous estimates suggest that this results in an underestimate of 35% of the costs. ^[[21]](#endnote-21)^

#### Classifications of clinical conditions and drugs categories

Diagnoses in secondary care are coded using ICD-10 codes and read codes are used in General Practice. England does not use ATC codes for pharmaceutical data, but rather the NHS Dictionary of Medicines and devices and the British National Formulary.

## Germany

### Health Care System1

#### Coverage, benefit package & payer structure for the study year (2012)

There are two systems that provide health insurance, which is required for every citizen, statutory health insurance (SHI) and private health insurance (PHI). For employed citizens there is a ceiling (52.200 € in 2013) which allows them to voluntarily be PHI-insured. Otherwise they and their nonearning dependents are covered by SHI. For some public sectors like police and military members there are special programs. Statutory health insurers levy a compulsory contribution which consists of a percentage of gross wages up to a ceiling (47.250 € in 2013. PHI companies calculate an individual risk-related premium. Citizens may choose their respective SHI or PHI freely.

SHIs cover all necessary health care services: inpatient and outpatient hospital care, physician services, mental health care, dental care, optometry, physical therapy, prescription drugs, medical aids, rehabilitation, hospice and palliative care, as well as sick leave compensation (in general after more than six weeks of sick leave. In the time before employers have to cover the salary payments) and certain preventive services. Preventive services under SHI include regular dental checkups, child checkups, basic immunizations, checkups for chronic diseases, and cancer screening at certain ages. SHIs are allowed to cover additional services such as travel vaccination or sport courses to gain competitive advantages over other SHIs. Home care is covered by long-term care insurance (LTCI).

#### Delivery system

Outpatient physicians – general practitioners (GPs) and specialists – who get reimbursed by SHI are by law mandatory members of regional associations (Kassenärztliche Vereinigungen) that negotiate collective contracts with SHIs. These regional associations of physicians function as financial intermediaries between the SHIs and the physicians in ambulatory care and have to provide a service guarantee for the ambulatory care provision. Physicians typically work in their own private practices with one or more doctors. Hospitals also negotiate collective reimbursement contracts with SHIs. Individuals have free choice among GPs, specialists, hospitals and other health care providers.

#### Reimbursement

SHI-accredited outpatient physicians are generally payed on a fee-for-service (FFS) basis, but reimbursements are budgeted to covering a predefined maximum number of patients per practice and reimbursement points per patient, setting thresholds on the number of patients and of treatments per patient for which a physician can be reimbursed. For the treatment of private patients, GPs and specialists also get an FFS, but the private tariffs are usually higher than the tariffs in the SHI uniform fee schedule. Hospitals are reimbursed by the German DRG (Diagnoses Related Groups) fee per case system, but also some budget restraints are implemented here. Other health care providers are also mostly payed on a FFS or fee per case basis.

#### Long term care and social services

Like health insurance LTCI is mandatory and offered by mostly the same statutory and private insurances. The contribution (2.05 % of gross salary plus 0.25% for insured persons without children in 2013 ^[[22]](#endnote-22)^) is shared between employers and employees. SHIs cover medical services and palliative care in a hospice. If the SHI Medical Review Board has determined a care level Hospice care is partly covered by LTCI.

### Data Source

We used person-level claims data from 2013 from two German statutory health insurers from the Kinzigtal region. This dataset contained all claims based on all covered services within the German Healthcare Insurance Act (SGB V, see description above). We excluded beneficiaries who were not enrolled at the insurer throughout the whole year except of those who died or were born during the year. In addition, beneficiaries who did not live in the Kinzigtal region during the whole year were excluded. Our overall analytic sample consisted of **30.355** individuals (after exclusions), **1.518** of whom were in the top 5 percent of spending.

The dataset contained several beneficiary characteristics, including gender, date of birth, date of death. For all types of care the insurance company provided payment infos. For hospital care the diagnosis, specialism, procedures and DRGs for reimbursements of hospital care as registered were provided. For mental health care the diagnosis and costs were registered. For pharmaceutics the cost and the pharmaceutical classification according to the Anatomical Therapeutic Chemical (ATC) system were provided. Finally, the fees for General Practitioners, specialists, physiotherapist, ambulance transport and all other remaining costs as reimbursed by the health insurance company, such as health care in foreign countries, were added. Not included are costs for services that are not covered, certain co-payments (e.g. for drugs), and all costs that are not patient/individual-related like refunds, discount agreements, special integrated care contracts without patient orientation and hospital investment costs for infrastructure because they are paid individually by the states.

### Study population

We only used data from the Kinzigtal region that were provided by the two statutory health insurers. This region is more rural and the insured persons are on average one year older than in whole Germany.

Patients who live in long-term care homes are included in the data but costs that are related to their long-term care are not in the data.

### Limitations and adjustments

#### Payment adjustment

Actual costs provided by insurers are used. These are in general the net-costs for the insurers, excluding co-payments. For pharmaceuticals only gross-costs are provided, as rebates and co-pays are not available on prescription level. For outpatient physician (GPs, specialists) costs, also gross costs are used. In Germany in the outpatient sector outpatient physician costs are budgeted, if they go beyond a certain threshold (EBM- remuneration system). Outpatient physician and outpatient medication costs for insurers may therefore be overestimated to a certain degree.

#### ICHA-HC Functions

| Category | National database (ICHA-HC functions) |
| --- | --- |
| Inpatient | HC 1.1 Inpatient Curative Care and HC 1.2 Day Curative Care |
| Outpatient | HC 1.3 Outpatient Curative Care |
| Rehabilitation | HC 2 |
| Drugs | HC 5.1 Pharmaceuticals. NB only outpatient prescription medication |
| Other | Medical goods, Medical care abroad, Patient transport |

#### Classifications of clinical conditions and drugs categories

Curative care data are coded using ICD 10 and pharmaceutical data were amended with ATC code using the provided pharmaceutical registration number in the analyses ATC level 2 was used.

#### Categorizing mental health costs

Mental health is identified by ICD and ATC Codes. An insured person is categorized as "mental health" if more than 50% of all healthcare costs are directly related to mental health. Outpatient costs could not be directly related to the diagnoses per case and thus were divided equally to the single diagnoses.

## Japan

### Health care system

*Payer structure, coverage, reimbursement, and delivery system*

The financial source of the National medical expenditure comprises three components, namely, social insurance system (49%), public fund including tax subsidies (39%) and out-of-pocket (OOP) payment (12%) (FY2012). ^[[23]](#endnote-23)^ Japanese health insurance system achieved universal health coverage in 1961, and basically all inhabitants are enrolled in a social health insurance plan which is based on employment and residential status. This system allows all participants to choose any healthcare facilities anytime nationwide (i.e., no gate-keeping system), and to receive necessary and qualified medical services with relatively low OOP payments. ^[[24]](#endnote-24),^^[[25]](#endnote-25),^^[[26]](#endnote-26)^

Majority of healthcare facilities are privately owned (70% of hospitals and 94% of clinics), and payment for out-patient is traditionally based on fee-for service system with macro cap. However, price-setting of healthcare costs are controlled by Ministry of Health, Labour and Welfare (MHLW) under a uniform fee schedule which is set every two years. With regard to payment for inpatient, prospective payment system known as the Diagnosis Procedure Combination Per-Diem Payment System (DPC/PDPS) has been implemented in 2003, and 55% of all acute care hospitalization were covered by this system.^24,^^[[27]](#endnote-27)^ Citizens are required to pay certain insurance premiums and co-payments (0-30%). The co-payment rate is as follows: 0-5 years 20%; 6-69 years 30%; 70–75 years 20%; and ≥75 years 10% (2017). Further, there are monthly and annual cap on the OOP payments to individuals.

Healthcare services covered by this system are more than 5000 medical procedures, dental care and drugs. Scope of the coverage are reviewed by MHLW every two years. Non-diseases including normal delivery, cosmetic purposes are not covered and patients are required to pay the full amount. ^24^

The two major insurance schemes in Japan are Employees’ Health Insurance and the National Health Insurance (NHI). The former system mainly covers public servants or those who work in companies, while the NHI covers individuals not working in companies (e.g. the self-employed, retired, unemployed, and their families). As a whole, there are more than 3000 insurers, and the premium rate largely differ among insurance scheme in Japan. In addition, Long Life Medical Care System is another major insurance scheme, which separated the elderly aged 75 years and older from the existing insurance system and were implemented in 2008 as a health care reform. Disabled individuals aged 65–74 years are also included in this scheme. ^24,27^

*Long term care services*

Mandatory Long-term care insurance (LTCI) system (Kaigo hoken) have been introduced since 2000 and covers the informal care of the population aged 40 and over. Medical insurers collect premiums from insured person aged 40-64 and pay these premiums as long-term care levy to the Social Insurance Medical Fee Payment Fund.

*Mental health care*

Mental health care is traditionally unique to long duration of hospitalization, such as 281 days on average in 2014.^[[28]](#endnote-28)^ Due to the governmental historical policy and price setting, 90% of psychiatric beds are privately owned. Moreover, to be discharged from hospitals is still challenging for patients, families and communities in Japan.

### Data Source

National health insurance claims data from 2 prefectures between April 2014 and March 2015 were used for analysis. This dataset comprised reimbursement claims submitted from healthcare providers to the NHI and Long Life Medical Care System, two major insurance payers in Japan. LTCI claims data which contains the elderly living at home or in nursing care facilities are not included in this study. However, we have to note that long-term care beds which provides medical care are partly included in the former two insurances.

The dwelling population in the two target prefectures was approximately 4 million, which means 3% of the total population in Japan (127 million) in FY2014. The former two major insurance claims data is estimated to cover 70-80% of the whole claims data of the dwelling population,^[[29]](#endnote-29)^ and when considering age group, ≥ 75 years are covered 96%, 65-74 years 77%, and 0-64 years 25% (FY2014).

### Study population

All patients who received any kind of health services and are registered in the former 2 insurances are included in this study. After excluding inconsistent records, overall analytic sample comprised 72,365 patients in the top 5 % and 14,472 patients in the top 1 % of spending.

### Limitations and adjustments

#### Study population

Our sample population covers most of the older generation of the dwelling population but less with the younger generation. However, since Japan is a leading super-aging society that 26% of the whole population were aged 65 and over (FY2014), and these population tend to receive more medication, our sample would be representative of national population in a certain degree and comparable with especially age 65 or above population in other countries.

#### Payment

Actual costs provided by insurers are used for analysis. These are costs for the insurers and contains co-payments. However, we could not identify the individual OOP payment as this information are not included in the database separately.

#### Classifications of clinical conditions and drug categories

We used the drug code of National Health Insurance Drug List in this study which is generally used in Japan instead of ATC codes. Within the index drug code, the first 2 digits includes 35, whereas the first 3 digits includes 142. We used the first 3 digits and calculated the number of unique code as to be comparable with ATC code level 3 which includes 93 classifications in other countries.

Regarding the average total number of nights spent in the hospital, we found that mental health care beds and long-term care beds included in the medical care were major driving force of the longest length of stay of our country in this study.

## The Netherlands 1

### Health care system

#### Coverage, benefit package & payer structure for the study year (2012)

Medical care is financed under the Health Insurance Act (HIA), a statutory health insurance system, with universally-mandated private insurance (national exchange). This means that all residents (including residents of long-term care institutions) are required to purchase statutory health insurance from private insurers. Residents choose their health plan in December for the duration of, at least, a full calendar year. The four largest insurance conglomerates account for 90 percent of all enrollees.

Health insurers are legally required to provide a standard benefits package including, care provided by general practitioners (GPs), hospitals, and specialists; dental care through age 18 (coverage after that age is confined to specialist dental care and dentures); prescription drugs; physiotherapy through age 18; basic ambulatory mental health care for mild-to-moderate mental disorders, including a maximum of five sessions with a primary care psychologist; and specialized outpatient and inpatient mental care for complicated and severe mental disorders. In case the duration of inpatient specialized mental health care exceeds one year, the last of these was financed under the Exceptional Medical Expenses Act (AWBZ). Some treatments, such as general physiotherapy and pelvic physiotherapy for urinary incontinence, are only partially covered for some people with specific chronic conditions, as are the first three attempts at in vitro fertilization. Apart from the basic benefit package, most of the inhabitants have complementary coverage for benefits excluded from the statutory package such as dental care, alternative medicine, physiotherapy, glasses, hearing aids, contraceptives and copayments.

The curative health system is financed through a combination of community-rated premiums set by each insurer and income-related contributions. The government provides healthcare allowances for people with low incomes. All insured over age 18 are required to pay a deductible of €220 (in 2012). However, some services (primarily GP visits) are exempted.

#### Delivery system

The general practitioner has a gatekeeping function for specialist and hospital care. Most citizens are registered with a GP, but registration is not formally required and there is no restriction on switching between GPs. GPs also provide after hour care, and some mental care (for mild-to moderate mental disorders) is integrated in GP offices. GPs generally refer complicated and severe mental disorders to specialized mental health care institutions, psychologists, or independent psychotherapist.

#### Reimbursement

Reimbursement for primary care is a mix of capitation and fee-for-service. In addition, GPs may negotiate bundled payments and pay-for-performance bonuses with insurers. Hospitals are reimbursed based on a DRG-like system (case-based) that include physician costs and in- and outpatient services.

#### Long term care and social services

Long-term care was provided both in institutions and in communities. It was financed by the AWBZ in 2012 and made up 38 percent of the total health care budget. Most palliative care is integrated into the regular health system. Although the number of hospices and palliative units has been growing for a number of years, still less than 5 percent dies in a hospice.

### Data Source

We used claims data from one of the Dutch insurance companies in 2012. This dataset contained all claims based on all covered services within the Healthcare Insurance Act (see description above). The dataset contained several beneficiary characteristics, including gender, date of birth, date of death~~.~~ Socio-economic status was added based on the 4-digit postal code of the beneficiary. This compound measure is provided by The Netherlands Institute for Social Research and based on 1) mean annual income per household, 2) the percentage of households with a low income and 3) the percentage of households with a low education.^[[30]](#endnote-30)^

For all types of care, except hospital care (inpatient and outpatient) and pharmacy data, the insurance company provided actual payments. For hospital care, the insurer provided codes for the diagnosis, specialism and a DRG-like product (DOT). To obtain cost data, we linked this information to standardized claims fees provided by the Dutch Health Care Authority. ^[[31]](#endnote-31)^ Standardized prices were used for pharmacy claims.

### Study population

The insurer that provided the data is one of the four main nationally operating insurance conglomerates in the Netherlands. The market share was about 20% of the population, covering 3.3 million beneficiaries. This population included people who live in long-term care residencies. The insurance company selected the top 5% of their most cost incurring beneficiaries in 2012 (N=166.291) based on all covered services within the basic benefits package (see description above). We have no indication that the insurer’s population differed significantly from the total population in the Netherlands.

### Limitations and adjustments

#### ICHA-HC Functions

| Category | National database (ICHA-HC functions) |
| --- | --- |
| Inpatient | HC 1.1 Inpatient Curative Care |
| Outpatient | HC 1.2 Day Curative Care and HC 1.3 Outpatient Curative Care |
| Rehabilition | HC 2 |
| Drugs | HC 5.1 Pharmaceuticals. NB only outpatient prescription medication + so called ‘expensive drugs’ used in-hospital (for example cancer drugs, anti TNF alpha) |
| Other | Durable medical goods, Medical care abroad, Patient transport, non-specified mental health costs |

#### Classifications of clinical conditions and drugs categories

The Dutch coding system for claims data does not include ICD10-codes. For hospital care (in- and outpatient), we mapped the diagnosis and specialism codes as provided in the claims data set into ICD 10 codes using a mapping table provided by the Dutch Health Authority. In addition, secondary mental hospital claims were summed with hospital claims to ICD10-chapter F (mental and behavioral disorders). Unfortunately, it was not possible to map other types of care to ICD10-codes. Thus, the results on the variable ‘number of unique ICD10-Chapters’ can only be displayed for inpatient and specialized outpatient care and secondary mental care. Pharmaceutical data came with ATC code, in the analyses, ATC level 2 was used.

## Spain

### Health care system

The Spanish National Health System (SNS) builds upon the foundations of a ‘Beveridge’ model, and is composed of 17 twin sub-systems, as many as Autonomous Communities comprise the Spanish quasi-federal State. In the statutory SNS, insurance is mandatory with coverage virtually universal, mainly funded from taxes and predominantly provided within the public sector, with strong gatekeeping role played by specialized primary care physicians serving the entire population. Provision is free of charge at the point of delivery, with the exception of pharmaceuticals and some ancillary goods, where copayment is set considering a maximum ceiling of monthly payment, fixed according to annual household income.

Public provision in the SNS plays a prominent role and, in general, the Health Departments of each Autonomous Community purchase the services to another public body, closely linked to the former, so called Regional Health Service. The latter is the administrative structure that runs all inpatient and outpatient health care centers. Generally, the health department annually contracts (and budgets) the service with the RGH that, in turn, negotiates global annual contracts with its integrated providers, primary care centers and hospitals and allocates lump-sum budgets to them. Additionally, the ACs’ Health Departments may contract extra services to private providers, usually hospitals that generally play a subsidiary role.

A typical patients’ pathway will work as follows: In general, upon the onset of symptoms, individuals will visit the primary care (PC) doctor with whom they are registered. The episode can be resolved by direct prescription or recommendation, follow-up in the same premises, or will require further diagnostic procedures. In this case, the patient will be given an appointment for testing (i.e. a wide range of lab and X-ray tests) and another one to return to the PC office where the results will be available in a few days. The PC’s clinical judgement will determine whether referral to specialized care is required, although Spanish patients will mostly have their problem solved at this care level.

When referral is necessary, the patient will be provided with an appointment for the corresponding specialist, usually based in outpatient premises. Based on the PC doctor referral report and their clinical assessment, the specialists will decide on the need for further testing or inpatient procedures, or will prescribe the treatment and, either send the patient back to PC or arrange follow-up visits. Once the specialist discharges the patient, a report is sent to the referring PC doctor.

If the decision involves inpatient care, the patient will be admitted to the corresponding hospital service, the timing depending on the waiting time for the corresponding procedures (diagnostic or surgical). Once discharged from the hospital, the patient is given an appointment for ambulatory follow-up, either at the specialized ambulatory services hierarchically linked to the hospital department, or at PC level, where PC physicians and nurses will take over upon the prescriptions recorded in the clinical report at discharge. Should the patient's condition be considered chronic at any of the previous stages, or at the time of their discharge from specialized care, the PC physician would require the intervention of the PC nurse for support and coordination of continuous follow-up and, depending on the social situation, for assessment by social services. If required, patients can be referred to rehabilitation services, either by the specialist or by the PC doctor.

Besides this regular pathway, two emergency mechanisms are available for patients to freely walk in: primary health care centers and hospital emergency wards. The former varies according to supply organization and expected demand: in rural disperse areas there is a 24-hour service, while in urban highly populated areas, PC services are complementary to hospital A&E wards, with a timetable covering 3pm to 8pm. In turn, hospital emergency departments offer 24/7 services. In addition, if the patient’s condition does not allow for walking in, patients can demand a home visit by the primary health care service, or use the emergencies call centre to request a mobile emergency team.

### Study Population

The 65,179 patients composing the Spanish cohort represent the universe of individuals at the 5% high-cost top within the 1.3 million lives that, in 2015, where insured by the Health Department in Aragon, one of the 17 Autonomous Communities comprising the statutory SNS in Spain.

### Data sources

Data come from a secure anonymied health information data-lake (SAHID), composed of different health care datasets covering the whole Aragonese population, and curated by the Institute for Health Sciences in Aragon (IACS), public institution organically linked to the Department of Health in Aragon. The different data sources are linked using the unique individual identifier (UII) assigned to each individual, when registers in the statutory SNS. For the purposes of this study, several data sources composing the SAHID have been deterministically linked; so: a) Master file of Insured Population (namely, BDU), which includes the information on the primary care centre where individuals are assigned to, demographic information, level of copayment, and the administrative situation (e.g., whether the individual remains as an insuree, has changed residence or has died); b) Database of Primary Care contacts (namely, OMI-AP) that has been used to elicit the primary case visits, and whether these visits (primary or consecutive) were served in the health centre, either by a doctor or a nurse, or at home, as an outreach activity; c) Database for outpatient specialized visits (namely, HIS) where contacts were differentiated between primary and subsequent visits; d) Database for A&E activity (namely, PCH-URG) where contact in hospitals emergency departments are registered; e) hospital admissions (namely, CMBD) which includes administrative (e.g., length of stay) and clinical information (e.g., diagnoses and procedures affecting the episode), from each of the hospital episodes yielded in acute or rehabilitative public hospitals; and, e) Database for electronic prescriptions, that registers drug dispensations and the retailing price for each of them.

Given the lack of full-costing methodology in the Spanish SNS, the allocation of unit prices to patients’ contacts required the use of additional instrumentation; so, a) in the case of primary care contacts (primary and consecutive), outpatient specialized visits (primary and successive) and A&E contacts, unit prices were extracted from the public tariffs used to reimburse public providers for the assistance delivered to a patient from a different Autonomous Community; b) in the case of hospital admissions, adjusted-unit prices were set upon the DRG grouping of hospital episodes and the actual hospital expenditure, except capital investments; and c) in the case of drugs prescriptions, the actual retailing price, including both the contribution of the SNS and the patients’ copayment, was allocated to each drug dispensed to the patients.

### Limitations and adjustments

The data sources included in the study cover the whole experience of the Aragonese population along 2015, and the coverage of each of the data sources used in the study is virtually universal. Moreover, as every single insuree holds a unique individual identifier the linkage capacity across data sources is unambiguous so that, all existing contacts in 2015 have been captured and included as part of the identification of the 5% high-cost Spanish cohort.

Price allocation could be an issue as there is no full costing methodology, particularly in the case of hospital episodes where a DRG adjusted-price has been allocated. On the other hand, there are some costs that might be underrepresented, as for example, lab or x-ray testing prescribed in primary care, transportation or dental care, which in Spain is chiefly provided privately. Nevertheless, the overall 2015 expenditure estimated with the different methodologies used in this work (public tariffs for outpatient contacts, adjusted-price for hospital episodes and retailing price for drug dispensations) accounts for the 98.3% of the actual expenditure consolidated in the National Health Accounts System for 2015.

## United States

### Main data source

We obtained 2013 and 2014 annual person-level and event-level files from the Medical Expenditure Panel Survey (MEPS).^[[32]](#endnote-32)^ We combined these into a single “average” file between the two. This offered a larger sample size with which to conduct our analysis. Our overall analytic sample consisted of **71,815** observations, weighted to be representative of the 317 million population. Of these observations, 2,842 were in the top 5 percent of adjusted spending, representing 15.8 million people in the population.

### Limitations and adjustments

*Payment Adjustment*

Because the spending numbers available in MEPS are actual payments, differences in unit prices paid by each payer in the U.S. (Medicaid vs Medicare vs Private Insurance) could potentially contribute to misclassifying some individuals as high-cost, while missing others. In order to address this, we standardized payments at the clinical classification code (CCC) level to Medicare prices. In order to do so, we ran a linear regression with payment as the outcome and CCC as the predictor. (Note: In order to identify the primary payer, we looked at the source of payment making up the biggest share of total payment for the event.) We then used the -margins- command in Stata to adjust these values to the Medicare rates reported in MEPS. We did not adjust drug payment data, nursing home data, ancillary payments, or dental payments. Drug spending was not adjusted because MEPS does not capture the primary variation between public and non-public payers – the rebate paid by manufacturer to an insurer.

*Mental Health Spending*

In order to more completely identify mental health spending in a physician’s office by examining the CCS diagnosis code reported in MEPS, rather than simply restricting our measure to mental health professionals.

*Identifying ATC Codes*

MEPS does not include ATC codes for the drug spending reported. However, we consulted with Fabricio Kury, National Institutes of Health, who has developed a script to match NDC codes with appropriate ATC codes. Using this script resulted in **94.7%** of drug event observations having an identifiable ATC code.

### Out-of-pocket spending

Out-of-pocket costs for an average high-cost patient were about 8 times higher in the US than in the Netherlands and Germany. For the other countries, data of out-of-pocket costs were not available. However, this varied from $631 for patients considered “dual-eligible” for Medicare and Medicaid, to up to $3140 for patients with Medicare and private insurance. Out-of-pocket spending was highest for American uninsured patients, about $6013

#### **eTable 5**: Out-of-pocket spending across payers

|  | **Top 5%** | **Top 1%** |
| --- | --- | --- |
| **<65 Any Private** | $3,110 | $3,750 |
| **<65 Any Public** | $1,316 | $2,017 |
| **<65 Uninsured** | $6,013 | $5,372 |
| **65+ Medicare Only** | $2,625 | $2,754 |
| **65+ Medicare + Private** | $3,140 | $5,677 |
| **65+ Medicare+Other Public** | $631 | $589 |
| **No Medicare, any public/private** | $1,157 | $1,483 |

1. Mossialos E, Djordjevic A, Osborn R, Sarnak D (eds). International Profiles of Health Care Systems [Internet]. 2017 [cited 2017 Jul 19]. Available from: http://www.commonwealthfund.org/~/media/files/publications/fund-report/2017/may/mossialos_intl_profiles_v5.pdf [↑](#endnote-ref-1)
2. <http://www.euro.who.int/en/about-us/partners/observatory/publications/health-system-reviews-hits/full-list-of-country-hits> [↑](#endnote-ref-2)
3. Schreyögg J, Stargardt T, Velasco-Garrido M, Busse R. Defining the “health benefit basket” in nine European countries: Evidence from the European Union Health BASKET Project. Eur J Heal Econ. 2005;6(SUPPL. 1):2–10. [↑](#endnote-ref-3)
4. Wismar M, Palm W, van Ginneken E, Busse R, Ernst K, Figueras J. The Health Service Initiative: supporting the construction of a framework for cross-border health care. Cross-border Heal Care Eur Union Mapp Anal Pract policies. 2011;1–22. [↑](#endnote-ref-4)
5. van der Wees PJ, Wammes JJG, Westert GP, Jeurissen PPT. The Relationship Between the Scope of Essential Health Benefits and Statutory Financing: An International Comparison Across Eight European Countries. Int J Heal Policy Manag [Internet]. 2015;5(1):13–22. Available from: http://ijhpm.com/article_3094_629.html [↑](#endnote-ref-5)
6. Velasco-garrido M, Stargardt T, Busse R, Velasco-garrido M. Identification of health baskets in nine EU countries. 2006 [↑](#endnote-ref-6)
7. Bradley EH, Sipsma H, Taylor LA. American health care paradox - high spending on health care and poor health. Qjm An Int J Med [Internet]. 2017 [cited 2017 Jul 13];110(2):61–5. Available from: https://oup.silverchair-cdn.com/oup/backfile/Content_public/Journal/qjmed/110/2/10.1093_qjmed_hcw187/1/hcw187.pdf?Expires=1500076188&Signature=EsyG6TBCu54RbWgwaQejS~6wv1o7bwCJvF3ypjn3lJ7lbEF8NlN2E8TTaTuA67iNOjtR3MUJnkxKomI2rJHVKmdzhUOT-hnp88hj5zG9MkfzVxA4 [↑](#endnote-ref-7)
8. OECD, Eurostat, WHO. A System of Health Accounts [Internet]. 2011 [cited 2017 Jun 12]. Available from: http://www.who.int/health-accounts/methodology/sha2011.pdf [↑](#endnote-ref-8)
9. World Health Organization. WHO | International Classification of Diseases [Internet]. WHO. World Health Organization; 2017 [cited 2017 Jul 21]. Available from: http://www.who.int/classifications/icd/en/ [↑](#endnote-ref-9)
10. Source: http://www.health.gov.on.ca/en/ministry/hc_system/ [↑](#endnote-ref-10)
11. Wodchis WP, Bushmeneva K, Nikitovic M, McKillop I. Guidelines on Person-Level Costing Using Administrative Databases in Ontario. *Working Paper Series.* Vol 1. Toronto: Health System Performance Research Network; 2013. [↑](#endnote-ref-11)
12. Peter Davies The concise NHS handbook 2013-2014. NHS CONFED, London UK. [↑](#endnote-ref-12)
13. https://www.google.co.uk/url?sa=t&rct=j&q=&esrc=s&source=web&cd=15&ved=0ahUKEwjMn8zmk_bYAhVWF8AKHV_gC7MQFghzMA4&url=http%3A%2F%2Fresearchbriefings.files.parliament.uk%2Fdocuments%2FCBP-7206%2FCBP-7206.pdf&usg=AOvVaw0rTNSRvuQg93r65eG3fCVT [↑](#endnote-ref-13)
14. https://www.cprd.com/Home/ [↑](#endnote-ref-14)
15. Herrett E, Gallagher AM, Bhaskaran K, et al. Data Resource Profile: Clinical Practice Research Datalink (CPRD). Int J Epidemiol2015;356:827-36. [doi:10.1093/ije/dyv098](http://dx.doi.org/10.1093/ije/dyv098) [pmid:26050254](http://www.ncbi.nlm.nih.gov/pubmed/?term=26050254) [↑](#endnote-ref-15)
16. http://www.pssru.ac.uk/project-pages/unit-costs/unit-costs-2017/ [↑](#endnote-ref-16)
17. https://www.gov.uk/government/publications/nhs-national-tariff-payment-system-201617 [↑](#endnote-ref-17)
18. https://www.gov.uk/government/collections/nhs-reference-costs [↑](#endnote-ref-18)
19. https://www.cprd.com/ObservationalData/CostData.asp [↑](#endnote-ref-19)
20. Personal Social Services Research Unit (PSSRU) Costs of Health and Social Care 2015. https://www.pssru.ac.uk/project-pages/unit-costs/unit-costs-2015 [↑](#endnote-ref-20)
21. Figure 2.2 Roberts A, Marshall L and Charlesworth A (2012) A decade of austerity? The funding pressures facing the NHS from 2010/11 to 2021/22. Research report. Nuffield Trust. [↑](#endnote-ref-21)
22. https://www.tk.de/tk/beitraege-grenzwerte/pflegeversicherung/660130 - 05.10.2017 [↑](#endnote-ref-22)
23. Annual Health, Labour, and Welfare Report 2015. http://www.mhlw.go.jp/english/wp/wp-hw9/dl/02e.pdf (accessed 20180215) [↑](#endnote-ref-23)
24. World Health Organization, Regional Office for South-East Asia. Japan health system review. Health systems in transition. Vol-8.No.1 2018. [↑](#endnote-ref-24)
25. Robertson R, et al. The social care and health systems of nine countries. The King’s Fund 2014. [↑](#endnote-ref-25)
26. Ikegami N, et al. Japanese universal health coverage: evolution, achievements, and challenges. Lancet 378:1106-1115, 2011 [↑](#endnote-ref-26)
27. Anderson G, Ikegami N. How can Japan’s DPC inpatient hospital payment system be strengthened? Lessons from the U.S. Medicare prospective system. A report of the CSIS global health policy center. 2011. [↑](#endnote-ref-27)
28. OECD,2017c, Japan’s data; Ministry of Health, Labour and Welfare, 2017. [↑](#endnote-ref-28)
29. Fact-finding Survey on Provision of Medical Services FY2014. Only in Japanese. [↑](#endnote-ref-29)
30. Statusscores, available from: http://www.scp.nl [↑](#endnote-ref-30)
31. www.opendisdata.nl [↑](#endnote-ref-31)
32. https://meps.ahrq.gov/mepsweb/ [↑](#endnote-ref-32)
